# Influenza vaccination in patients with multiple sclerosis is possible with some considerations

**Published:** 2016-04-03

**Authors:** Seyed Mohammad Baghbanian

**Affiliations:** ^1^ Department of Neurology, School of Medicine AND Booalisina Hospital, Mazandaran University of Medical Sciences, Sari, Iran

**Keywords:** Multiple Sclerosis, Influenza, Vaccine

## Introduction

Influenza is a disease of particular concern for patients with multiple sclerosis (MS). Is influenza vaccine good for MS? 

**Types of influenza vaccine**

Two types of influenza vaccine are used. The first one includes inactivated (killed) influenza virus administered intramuscularly and the second, live, attenuated, virus administered intranasally via an aerosol sprayer.^1^ Live attenuated vaccine is not recommended for MS patients.

**Risk of MS onsets after influenza vaccination**

Studies of the onset of MS after influenza vaccination had very serious methodological limitation and did not report any association between influenza vaccination and the increased risk of MS in adults.^2^^-^^5^

**Influenza disease in patients with MS**

Two studies showed that the risk of influenza-related hospitalizations, mortality, morbidity and relapse increased in patients with relapsing-remitting MS (RRMS).^6^^,^^7^

**Influenza** **‎** **vaccination and relapse**

It seems that influenza vaccination has a protective effect on MS and does not seem to exacerbate or deteriorate neurological status.^7^^,^^8^ Nevertheless, there is a small case series reporting relapses within 3 weeks of simultaneous H1N1 and seasonal vaccination.^9^

**MS drugs and influenza vaccination**

Corticosteroid: Corticosteroids did not prove to impair the immune response following influenza vaccination.^10^

Interferon-beta (INF-β): Seasonal influenza vaccination is safe and effective in 90.9% and 93.0% INF-treated patients.^11^

Glatiramer acetate (GA): GA ‎may present a lower protection after influenza vaccination compared to healthy individuals.^12^

Mitoxantrone: Mitoxantrone can impair influenza vaccine immunogenicity and efficacy.^12^

Teriflunomide: The TERIVA study showed that influenza vaccination was sufficient in providing the considered protection in patients treated with teriflunomide.^13^

Dimethyl fumarate: There is not any data on dimethyl fumarate and influenza vaccination.

Fingolimod: Influenza vaccination in fingolimod-treated patients could be safe and protective but need a booster dose.^14^

Natalizumab: Influenza vaccination could be safe and protective in natalizumab-treated patients.^15^

Cytotoxic: Azathioprine-treated patients with systemic lupus erythematosus (SLE) had a diminished antibody response following influenza vaccination.^16^

Intravenous immunoglobulin (IVIG): The immunogenicity of live vaccines was impaired by IVIG for 6-12 months.^17^

Rituximab: In rituximab-treated patients, vaccination with inactivated vaccines might be effective.^18^

**Time of vaccination**

Following corticosteroid pulse therapy, it is recommended to delay vaccination for at least 2 weeks. In patients with MS treated with mitoxantrone and cyclophosphamide, it should be done between drug cycles. In immunosuppressive therapy, antibody testing is recommended 4 weeks following the vaccination and if the antibody titers failed to rise, revaccination should be kept in mind.^19^

## Notes:

**How to cite this article:** Baghbanian SM. Influenza vaccination in patients with multiple sclerosis is possible with some considerations. Iran J Neurol 2016; 15(2): 109-10.
